# Iterative Deep Neighborhood: A Deep Learning Model Which Involves Both Input Data Points and Their Neighbors

**DOI:** 10.1155/2020/9868017

**Published:** 2020-01-02

**Authors:** Rong Liu, Yan Liu, Yonggang Yan, Jing-Yan Wang

**Affiliations:** ^1^School of Economics and Management, Chongqing Jiaotong University, Chongqing 400074, China; ^2^New York University Abu Dhabi, Abu Dhabi, UAE

## Abstract

Deep learning models, such as deep convolutional neural network and deep long-short term memory model, have achieved great successes in many pattern classification applications over shadow machine learning models with hand-crafted features. The main reason is the ability of deep learning models to automatically extract hierarchical features from massive data by multiple layers of neurons. However, in many other situations, existing deep learning models still cannot gain satisfying results due to the limitation of the inputs of models. The existing deep learning models only take the data instances of an input point but completely ignore the other data points in the dataset, which potentially provides critical insight for the classification of the given input. To overcome this gap, in this paper, we show that the neighboring data points besides the input data point itself can boost the deep learning model's performance significantly and design a novel deep learning model which takes both the data instances of an input point and its neighbors' classification responses as inputs. In addition, we develop an iterative algorithm which updates the neighbors of data points according to the deep representations output by the deep learning model and the parameters of the deep learning model alternately. The proposed algorithm, named “Iterative Deep Neighborhood (IDN),” shows its advantages over the state-of-the-art deep learning models over tasks of image classification, text sentiment analysis, property price trend prediction, etc.

## 1. Introduction

### 1.1. Background

Deep learning has been proven to be a powerful tool for pattern classification problems and sensor studies [1–15]. A deep learning model usually has more than three layers, and by using multiple layers, the model extracts hierarchical features from the original data. In this way, an abstractive feature can be generated from the high-level layers. The deep learning model can release the problem feature engineering by learning effective features automatically. The feature engineering process of traditional machine learning models relies on the domain knowledge of feature engineers heavily, and it is time consuming and the designed features cannot be generated to other domains. However, compared to the traditional machine learning and feature engineering process, the deep learning can automatically find the features which are relevant to the learning problem and store the features in the neural units of multiple layers. For example,

- In the problem of face recognition, deep learning models, especially deep convolutional neural network (CNN), have been a popular model to extract high-level features for individuals [16, 17]. The deep CNN model is composed of multiple convolutional layers and max-pooling layers. The low-level convolutional layers use a sliding window and a group of filters to extract local simple features from the facial images; the filters are based on simple local patterns such as circle, square, and edges. The middle-level convolutional layers take the outputs of the low-level convolutional layers and extract the patterns of parts of faces, such as eye, nose, and mouth. Finally, in the high-level layers, the patterns of individual faces are generated. In this way, the key features of faces of individual are extracted.
- Meanwhile, for the problem of text categorization, deep learning models are also playing a key role. The most popular deep learning model is the long-short-term memory (LSTM) model [18, 19]. A deep LSTM model is also composed of multiple layers of LSTM, while each layer processes the input sequence by using a sliding neural unit. The input to the first layer is the sequence of tokens (represented by the word-embedding vectors). The sliding neural unit slides over the sequence and takes both a current instance and a memory of the previous instance as inputs and outputs both a response for the next layer and a memory vector for the next instances. For the deep LSTM model, the low-level layers extract features from the tokens, and the middle layers extract features for the phases, while the high-level layers extract features for the sentence/texts and the final layers present the text by semantic feature.

Some recent advancements in deep learning can be found in [20–22].

### 1.2. Motivation

The existing deep learning models have achieved great success in some problems of different areas, such as computer vision, natural language processing, speech processing, signal processing, and bioinformatics. However, up to now, most of the existing deep learning models hardly achieve satisfactory performance in many other machine learning applications, due to its strong limitation of the input of the model. A traditional deep learning model only takes the input sequence of instances of the input data point as input, but it ignores the other data points in the dataset. The assumption behind this model is that the data instances of an input data point are sufficient to predict its class label. However, in real-world applications, the input data instances themselves are not sufficient for the prediction. For example, in the problem of sentiment analysis, given an input sentence (data point) “*My paper has been accepted by the journal*,” it is difficult for the deep neural network to decide if the sentiment is positive or negative only from the tokens (instances).

**Figure 1:**
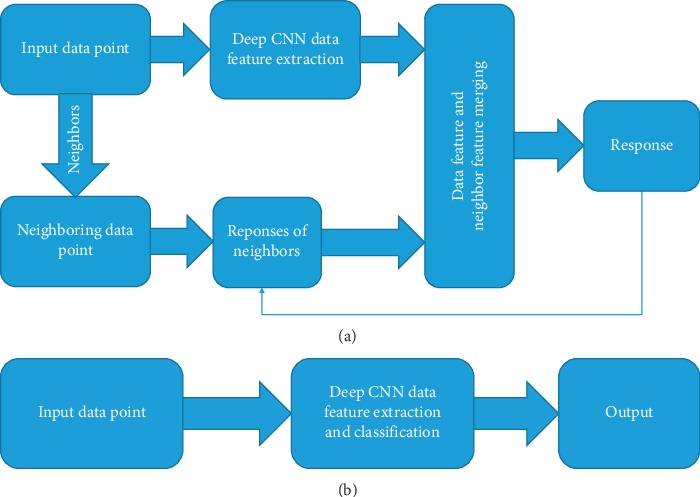
Illustration of using neighbors to enhance the prediction of deep learning and the traditional deep learning. (a) The proposed learning framework. (b) The traditional deep learning framework.

To solve this problem, we propose to leverage the neighbors of the input sentence and use their information to help the decision making of the sentiment of the input sentence. As an example, we find a similar neighboring sentence from the data set, “*Congratulations! Your paper is good enough to be accepted by the journal*,” and then use it to help the prediction of the input sentence. We design a model to take both the input sentence and the response of the neighboring sentence to do the prediction. Since the neighboring sentence contains some positive tokens such as “*Congratulations*” and “*good*”, the model can give a strong response to the positive sentiment. When taking this positive sentiment of the neighboring sentence into account, the model can also further decide that the input sentence has a positive sentiment. The thought of using neighbors' responses to enhance the prediction ability of the deep learning model is illustrated in Figure 1(a). As a comparison, we also show the traditional structure of deep learning model in Figure 1(b).

### 1.3. Our Contributions

In this paper, our contributions are given as three parts:

- Firstly, we proposed a new deep learning model for pattern classification problem. The key difference between our model and the traditional deep learning model is the input structure. The traditional deep learning model only takes the input instances of an input data point, but our model can take both the input data point and its neighboring points, to be specific, the classification responses of the neighbors, as the inputs of the model.
- Secondly, we proposed a new learning framework for the learning of the model parameters and the determination of the neighbors of the input data. We propose to learn the neighbors and the deep learning models in an iterative algorithm, while the neighbors with their classification responses and the deep learning model parameters are updated alternately. We update the nearest neighbors of each data point in the data space of the convolutional representation of the deep learning model and use the max-pooling of their deep learning model responses regarding different classes as the inputs of the deep learning model of the next iteration. An EM algorithm is developed to update the model parameters and neighbors.
- Remark: since our method learns the neighbors and model simultaneously, it is a local learning method. Loia et al. [23] developed an effective local learning method by merging a local weighted regression model and a fuzzy transform method (F-transform). The F-transform plays the role of the reduction method for the cardinality of the learning problem. Our local learning method is inspired by [23], but we solve a different problem of learning neighbors from deep CNN model.
- We evaluate the proposed joint deep learning and neighbor learning algorithm over several benchmark data sets, and the results show its advantage over state-of-the-art deep learning algorithms. We also study different properties of the proposed algorithm experimentally over the benchmark datasets and show the stability of the proposed method.

### 1.4. Paper Organization

In the rest of this paper, we introduce the proposed deep learning model and its learning process in Section 2. In Section 3, we evaluate the proposed algorithm by comparing it to state-of-the-art deep learning models and studying its properties experimentally. In Section 4, we conclude the paper with some potential future works.

## 2. The Proposed Method

In this section, we introduce the proposed deep learning model which takes both the input instances of a data point but also the neighboring data points' classification map as input. We will first introduce the inputs of the model, then the model structure, and finally the method to learn the parameters of the model.

### 2.1. Model Inputs

We assume we have a training set of *n* data points, and we deal with a multiclass classification problem of *K* classes. The training set is denoted as *𝒳*={(*X*_*i*_, *y*_*i*_)}_*i*=1_^*n*^, where *X*_*i*_ is the input data presenting the *i*-th data point and **y**_*i*_=[*y*_*i*1_,…,*y*_*iK*_]^*⊤*^ ∈ {1,0}^*K*^ is the class label vector of the *i*-th data point. *y*_*ik*_=1 if *X*_*i*_ belongs to the *k*-th class, and 0 otherwise.

To classify one data point in the training set, *X*_*i*_, the inputs of the model include two types of data as follows:

- Instance sequence: the first type of input is the instances of the data point itself as follows:

(1) $$\begin{array}{c} X_{i}=\left(x_{i1},\ldots ,x_{i\left|X_{i}\right|}\right), \end{array}$$

  where and **x**_*il*_ is the feature vector of the *l*-th instance of the *i*-th data point, and |*X*_*i*_| is the length of the sequence.
(i) Neighborhood classification map: the second type of the input is the neighborhood of *X*_*i*_ and the classification map of the neighborhood. The neighborhood dataset of *X*_*i*_ is denoted as *𝒩*_*i*_. To obtain the classification map of *𝒩*_*i*_, we first calculate the classification responses of the data points in *𝒩*_*i*_ for each class, then apply a classwise max-pooling operation, and finally concatenate the *K* maximum responses for *K* classes. We denote the classification response of a data point *X*_*j*_ ∈ *𝒩*_*i*_ regarding the *k*-th class as *p*_*jk*_ ∈ [0,1], and the classification map of *𝒩*_*i*_ is given as

(2) $$\begin{array}{c} p_{i}={\left[\underset{j:X_{j}\in N_{i}}{\max}p_{j1},\ldots ,\underset{j:X_{j}\in N_{i}}{\max}p_{jK}\right]}^{⊤}\in {\left[0,1\right]}^{K}, \end{array}$$

where max_*j*:*X*_*j*_∈*𝒩*_*i*__ *p*_*jk*_ is the max-pooling result of the classification responses over *𝒩*_*i*_ regarding the *k*-th class. The calculation of the classification responses *p*_*jk*_ will be introduced in the following sections.

For each input data point *X*_*i*_, according to the above description, we have two inputs as follows: (**x**_*i*1_,…, **x**_*i*|*X*_*i*_|_) and **p**_*i*_.

### 2.2. Model Structure

**Figure 2:**
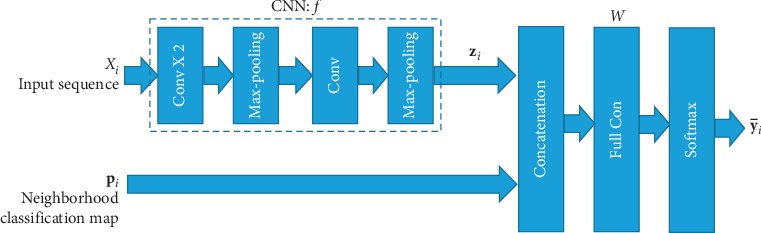
Overview of the proposed deep learning framework.

The overview framework of our mode is shown in Figure 2. This model is composed of a CNN model, denoted as *f*, one concatenation layer, one full-connection layer, and one softmax nonlinear transformation layer. The functions of these layers and the flow of the data in the model are introduced as follows:

- The input sequence of instances are firstly transformed to a vector of *d*-dimensional vector **z**_*i*_ ∈ *ℝ*^*d*^, by the CNN model, *f*, composed of three convolutional layers and two max-pooling layer:

(3) $$\begin{array}{c} z_{i}=f\left(X_{i}\right). \end{array}$$

(ii) Then, **z**_*i*_ is concatenated with the neighborhood classification map vector, **p**_*i*_ ∈ *ℝ*^*K*^, by the concatenation layer. The concatenated vector is denoted as $\left[\begin{array}{c} z_{i}\\ p_{i} \end{array}\right]\in {\mathbb{R}}^{d+K}$.
(iii) The concatenated vector is further reduced to a *K*-dimensional vector by the full-connection layer, and its connection weight matrix *W*=[**w**_1_,…, **w**_*K*_] ∈ *ℝ*^*d*+*K*^ × *K*, where **w**_*k*_ is its *k*-th column corresponding to the *k*-th class. The outputs of the full-connection layer is calculated as

(4) $$\begin{array}{c} W^{⊤}\left[\begin{array}{c} z_{i}\\ p_{i} \end{array}\right]={\left[w_{1}^{⊤}\left[\begin{array}{c} z_{i}\\ p_{i} \end{array}\right],\ldots ,w_{K}^{⊤}\left[\begin{array}{c} z_{i}\\ p_{i} \end{array}\right]\right]}^{⊤}. \end{array}$$

(iv) Finally, the outputs of the full-connection layer are normalized to probabilities over the *K* classes by the softmax activation layer, and the outputs are calculated as follows:

(5) $$\begin{array}{c} {\bar{y}}_{i}={\left[p_{i1},\ldots ,p_{iK}\right]}^{⊤}\in {\left[0,1\right]}^{K}, \end{array}$$

where

(6) $$\begin{array}{c} p_{ik}=\frac{\text{exp}\left(w_{1}^{⊤}\left[\begin{array}{c} z_{i}\\ p_{i} \end{array}\right]\right)}{\sum_{k\prime =1}^{K}\text{exp}\left(w_{k\prime }^{⊤}\left[\begin{array}{c} z_{i}\\ p_{i} \end{array}\right]\right)}, \end{array}$$

is the probability of *X*_*i*_ belonging to the *k*-th class and ${\bar{y}}_{i}$ is the output vector of the model. To decide the class of the given data point, *X*_*i*_, we choose the class with the largest probability:

(7) $$\begin{array}{c} y^{*}=\arg\underset{k=1,\ldots ,K}{\max}p_{ik}. \end{array}$$

#### 2.2.1. Model Parameter Learning

Learning problem modeling: In our model, there are two groups of parameters, which are the parameters of the CNN model, *f*, and the connection weight matrix *W*. To learn the parameters to fit the training data, we build a unified learning framework. In this learning framework, we propose to measure the classification error by the cross-entropy loss function and measure the complexity of the model by the squared *ℓ*_2_ norm of the parameters. Moreover, we proposed to minimize the classification error to improve the classification performance, and the complexity of the model to reduce the overfitting risk. The learning problem is modeled as a minimization problem, and the objective of the problem is given as follows:

(8) $$\begin{array}{c} O\left(W,f\right)=\overset{n}{\underset{i=1}{\sum }}\ell \left(y_{i},{\bar{y}}_{i}\right)+\frac{C_{1}}{2}\left({\left\|W\right\|}_{2}^{2}\;+\;{\left\|f\right\|}_{2}^{2}\right), \end{array}$$

where

(9) $$\begin{array}{c} \ell \left(y_{i},{\bar{y}}_{i}\right)=-\overset{K}{\underset{k=1}{\sum }}y_{ik}\log\left({\bar{y}}_{ik}\right)\\ =-\overset{K}{\underset{k=1}{\sum }}y_{ik}\log\left(\frac{\exp\left(w_{k}^{t⊤}\left[\begin{array}{c} z_{i}\\ p_{i} \end{array}\right]\right)}{\sum_{k\prime =1}^{K}\exp\left(w_{k\prime }^{t⊤}\left[\begin{array}{c} z_{i}\\ p_{i} \end{array}\right]\right)}\right), \end{array}$$

is the cross-entropy loss function for the *i*-th data point, ‖*W*‖_2_^2^ is the squared *ℓ*_2_ norm of *W*, ‖*f*‖_2_^2^ is the squared *ℓ*_2_ norm of the filters of the CNN model *f*, and *C*_2_ is the tradeoff parameter of the classification error term and the *ℓ*_2_ norm regularization term. The minimization problem is given as follows to learn the optimal parameters, *W*^*∗*^ and *f*^*∗*^, over the training set:

(10) $$\begin{array}{c} W^{*},f^{*}=\arg\underset{W,f}{\min}O\left(W,f\right),\\ \text{s}.\text{t}.\;z_{i}=f\left(X_{i}\right),\;\forall \;i=1,\ldots ,n. \end{array}$$

Please note that in our learning problem, we explicitly introduce a slick variable, the convolutional representation vector of the CNN model, **z**_*i*_, for each data point and impose it to be equal to the output of the CNN model, *f*(*X*_*i*_).

Problem optimization: it is difficult to solve the problem in (10) directly, because the classification map **p**_*i*_ itself is a function of *W*, *f*, and **p**_*j*:*X*_*j*_∈*𝒩*_*i*__ according to (2) and (5):

(11) $$\begin{array}{c} p_{i}={\left[\underset{j:X_{j}\in N_{i}}{\text{max}}\frac{\text{exp}\left(w_{1}^{⊤}\left[\begin{array}{c} z_{j}\\ p_{j} \end{array}\right]\right)}{\sum_{k\prime =1}^{K}\text{exp}\left(w_{k^{\prime }}^{⊤}\left[\begin{array}{c} z_{j}\\ p_{j} \end{array}\right]\right)},\ldots ,\underset{j:X_{j}\in N_{i}}{\text{max}}\frac{\text{exp}\left(w_{K}^{⊤}\left[\begin{array}{c} z_{j}\\ p_{j} \end{array}\right]\right)}{\sum_{k\prime =1}^{K}\text{exp}\left(w_{k^{\prime }}^{⊤}\left[\begin{array}{c} z_{j}\\ p_{j} \end{array}\right]\right)}\right]}^{⊤}\in {\left[0,1\right]}^{K}. \end{array}$$

Moreover, the parameters *W* and *f* are coupled. Thus, we adopt the EM algorithm to solve this problem. In an iterative algorithm, the parameters and the neighborhood classification map vector for each data point are updated alternately. In the M-step, we fix the classification map vectors **p**_*i*_|_*i*=1_^*n*^ and update *W* and *f* by minimizing the objective, while in the E-step, we fix the parameters *W* and *f* to update the neighborhood and the neighborhood classification map vectors. The E-step and M-step are introduced as follows:

- E-step: in the *t*-th iteration, we first use the CNN model *f*^*t*−1^ learned from previous iteration to update the convolutional vector of *X*_*i*_:

(12) $$\begin{array}{c} z_{i}^{t}=f^{t-1}\left(X_{i}\right). \end{array}$$

   Then, we use the convolutional representation vectors of the data points to update the neighborhood of each data point. The neighborhood of each data points are collected as its *k* nearest neighbors according to the *ℓ*_2_ norm distance collected:

(13) $$\begin{array}{c} N_{i}^{t}=k\;\arg\underset{X_{j}:j\neq i}{\min}{\left\|z_{i}^{t}-z_{j}^{t}\right\|}_{2}^{2}. \end{array}$$

  Then, we use the updated neighborhoods, *𝒩*_*i*_^*t*^|_*i*=1_^*n*^, the updated convolutional representation vectors, **z**_*i*_^*t*^|_*i*=1_^*n*^, the classification map vectors of previous iteration, **p**_*i*_^*t*−1^|_*i*=1_^*n*^, and the connection weight matrix of full-connection layer, *W*^*t*−1^, to update the classification map vectors of current iteration according to (11):

(14) $$\begin{array}{c} p_{i}^{t}={\left[\underset{j:X_{j}\in N_{i}^{t}}{\text{max}}\frac{\text{exp}\left(w_{1}^{t-1⊤}\left[\begin{array}{c} z_{j}^{t}\\ p_{j}^{t-1} \end{array}\right]\right)}{\sum_{k\prime =1}^{K}\text{exp}\left(w_{k\prime }^{t-1⊤}\left[\begin{array}{c} z_{j}^{t}\\ p_{j}^{t-1} \end{array}\right]\right)},\ldots ,\underset{j:X_{j}\in N_{i}^{t}}{\text{max}}\frac{\text{exp}\left(w_{K}^{t-1⊤}\left[\begin{array}{c} z_{j}^{t}\\ p_{j}^{t-1} \end{array}\right]\right)}{\sum_{k\prime =1}^{K}\text{exp}\left(w_{k^{\prime }}^{t-1⊤}\left[\begin{array}{c} z_{j}^{t}\\ p_{j}^{t-1} \end{array}\right]\right)}\right]}^{⊤}. \end{array}$$

(ii) M-step: in this step, we fix the classification map vectors of previous iteration, **p**_*i*_^*t*−1^|_*i*=1_^*n*^, and minimize the problem of (10) to obtain the solution of *W* and *f* for the *t*-th iteration:

(15) $$\begin{array}{c} W^{t},f^{t},Z^{t}=\arg\underset{W,f,Z}{\min}\left\{O\left(W,f\right)=-\overset{n}{\underset{i=1}{\sum }}\overset{K}{\underset{k=1}{\sum }}y_{ik}\log\left(\frac{\exp\left(w_{k}^{⊤}\left[\begin{array}{c} z_{i}\\ p_{i}^{t-1} \end{array}\right]\right)}{\sum_{k\prime =1}^{K}\exp\left(w_{k^{\prime }}^{⊤}\left[\begin{array}{c} z_{i}\\ p_{i}^{t-1} \end{array}\right]\right)}\right)+\frac{C_{1}}{2}\left({\left\|W\right\|}_{2}^{2}+{\left\|f\right\|}_{2}^{2}\right)\right\},\\ \text{s}.\text{t}.\;z_{i}=f\left(X_{i}\right),\;\forall \;i=1,\ldots ,n. \end{array}$$

  To solve this problem, we use the ADMM method. For each constraint **z**_*i*_=*f*(*X*_*i*_), we introduce a dual vector, ***θ***_*i*_ ∈ *ℝ*^*d*^, and rewrite the problem as follows:

(16) $$\begin{array}{c} \underset{W,f,Z}{\min}\left\{L\left(W,f,Z,\Theta \right)=O\left(W,f,Z\right)+\overset{n}{\underset{i=1}{\sum }}\frac{\beta }{2}\;{\left\|f\left(X_{i}\right)-z_{i}\right\|}_{2}^{2}+\overset{n}{\underset{i=1}{\sum }}\theta_{i}^{⊤}\left(f\left(X_{i}\right)-z_{i}\right)\right\}, \end{array}$$

where Θ=[***θ***_1_,…, ***θ***_*i*_] and *L*(*W*, *f*, *Z*, Θ) is the augmented Lagrangian function. According to the ADMM process, we iteratively update the primal variables **w**_*k*_|_*k*=1_^*K*^, *f* and **z**_*i*_|_*i*=1_^*n*^ to minimize the augmented Lagrangian using the gradient descent and use the gradient ascent method on the dual problem to update ***θ***_*i*_|_*i*=1_^*n*^:

(17) $$\begin{array}{c} w_{k}^{\text{new}}=w_{k}^{\text{old}}-\eta \nabla L_{w_{k}}\left(w_{k}^{\text{old}}\right),\;k=1,\ldots ,K,\\ f^{\text{new}}=f^{\text{old}}-\eta \nabla L_{f}\left(f^{\text{old}}\right),\\ z_{i}^{\text{new}}=z_{i}^{\text{old}}-\eta \nabla L_{z_{i}}\left(z_{i}^{\text{old}}\right),\;i=1,\ldots ,n,\\ \theta_{i}^{\text{new}}=\theta_{i}^{\text{old}}+\eta \nabla L_{\theta_{i}}\left(\theta_{i}^{\text{old}}\right),\;i=1,\ldots ,n, \end{array}$$

where ∇*L*_*x*_(*x*) is the subgradient function of *L* regarding *x* and *η* is the descent/ascent step.

## 3. Experiments

In this section, we evaluate the performance of the proposed deep learning method over several benchmark data sets.

### 3.1. Data Sets

We used the following datasets for the evaluation of the proposed method.

#### 3.1.1. SkyFinder Dataset

This dataset is for the problem of sky detection in hazy image [24, 25]. These data contain about 90,000 outdoor images, which are captured by 53 cameras. The number of images captured by each camera is of thousands. About 40% pixels of the images are the sky, and the problem is to predict sky from the image in pixel level. The input for the prediction of each pixel is the surrounding region of the target pixel.

#### 3.1.2. Multilingual Text Data Set

This dataset is composed of five subsets of texts [26]. Each subset is corresponding to a language. The five languages are English, French, German, Italian, and Spanish. The texts are of six different classes, which are C15, CCAT, E21, ECAT, GCAT, and M11. For each class of each language, the number of texts is no more than 5,000. The number of texts of each language varies from 12,000 to 30,000, and the number of unique tokens of each language varies from 11,547 to 34,279. The number of texts of each class also varies from 11,000 to 34,000. Each text is presented by a sequence of tokens, and each token is represented by a work-embedding vector trained by Glove algorithm [27]. Thus, each text is a data point, and a text is a sequence of work-embedding vectors. The problem is to predict the class label of a text from the sequence of word-embedding vectors.

#### 3.1.3. FERET Face Image Data Set

This dataset is an image data set of human faces [28]. It contains 13,539 images of 1,565 individuals. The images are of different ages, gender, and positions. Each image is of size 128 × 128 pixels. Each image is considered as a set of image patches, and thus, it is a 2 − D sequence of instances. The problem for this data set is to recognize the individual from a given face image.

#### 3.1.4. Property Price Data Set

This is a data set of time series of the nationwide building society housing price index (https://www.nationwide.co.uk). The time range of this data set varies from the year of 1973 to 2000. To generate the data points, we use a sliding window of one year to move over the time series. The time series within the window is considered as the input sequence of a data point. The overall trend of the following three months of the window is treated as the target of prediction. The trend is defined as “increase” if the price at the end of the three months is significantly higher than in the beginning, “decrease” if significantly lower, and “flat” otherwise. The problem is thus a three-class classification problem. To present each data point, we further use a smaller sliding window to splice the time series into a set of frames, and each frame is treated as an instance.

### 3.2. Experimental Setting

To conduct the experiments, we use the 10-fold cross-validation protocol to split the data sets into training sets and test sets. The entire data set is split into 10 equal-size subsets. Each subset is used as a test set in turn, and the other nine subsets are combined to construct a training set. The proposed algorithm is used to train the parameters of the deep learning models over the training set, and then, the model is applied to the test set. To classify one single data point in the dataset, we first find its nearest neighbors by comparing its convolutional representation against the convolutional representations of the data points in the training set and then use the deep neighbor classification map and its own convolutional representation to calculate a classification score to decide its classification result. The average classification rate over the ten test sets is used as the performance measure.

### 3.3. Compared Deep Learning Methods

Our model is the very first deep learning model which can take both input data and the neighbor information as input. Thus, there are no existing models for comparison. However, the contextual deep learning model uses the neighboring instances as contextual to enhance the feature extraction of each instance in the input data point. Note that the neighborhood information of the contextual deep learning model is at the instance level, while our Iterative Deep Neighborhood is at the data point level; this leverages more information than the contextual deep learning model. We compared the following contextual deep learning model to our methods:

- The Multicontext Deep Learning (MCDL) model was proposed by Zhao et al. [29]. This model was proposed to solve the problem of salient object presence in a low-contrast background, and it is based on the CNN model. Both global and local contexts are employed and jointly modeled in a unified multicontext deep learning framework of the model.
- The Multistage Contextual Deep Learning (MSCDL) model was proposed by Zeng et al. [30]. This model was proposed for the pedestrian detection problem, and it jointly trains multistage classifiers. Moreover, the local regions are used as contextual information to support the decision at the next stage. The deep learning model is trained in a stage-by-stage style.
- The Spatial Contextual Deep Learning (HCDL) model was proposed by Ma et al. [31]. This model is proposed for the hyperspectral image classification, and it uses both the feature and the spatial contextual information for the hyperspectral image classification. The spectral and spatial features are both learned by the deep learning framework to generate effective representations of the data.

### 3.4. Experimental Results

#### 3.4.1. Classification Results

**Figure 3:**
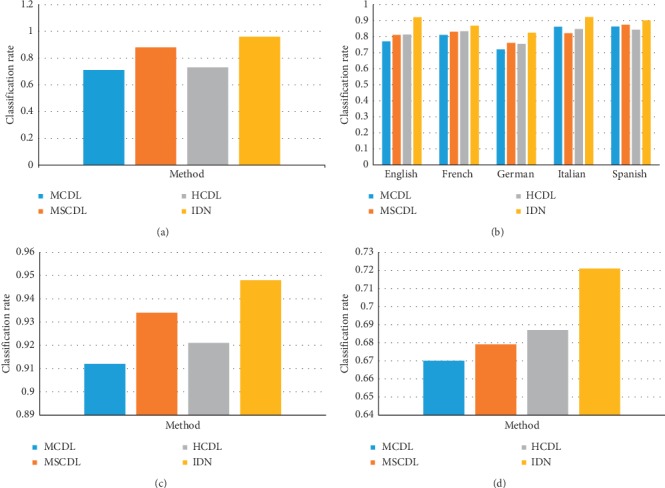
Comparison results over benchmark data sets. (a) SkyFinder. (b) Multilingual text. (c) FERET face. (d) Property price.

The classification results of the proposed method, Iterative Deep Neighborhood (IDN), and the compared methods are given in Figure 3. According to the results reported in Figure 3, we have the following observations:

1. Among all the data sets, our method obtains the best classification rate. This is a strong evidence for our claim that the neighbors help to build a more effective deep learning model for the problem of classification. For example, in experiments over the multilingual text data set, for the English language, all the other methods give classification results around 0.8, while IDN achieves as good performance as over 0.9.
2. This is not surprising since IDN, to classify one sentence, has the ability to explore the other neighboring sentences. Moreover, it can also refine the neighboring sentences according to the previous classification results. But for the other context-based deep learning models, they can only explore the neighboring words in the test sentences while ignoring the other sentences.
3. For the other methods, MSCDL and HCDL outperform the MCDL algorithm. However, it is not clear which one of MSCDL and HCDL performs better. In the SkyFinder and FERT face data sets, MSCDL obtains better accuracy, and in the property price data set, HCDL is a better solution.

#### 3.4.2. Convergence

**Figure 4:**
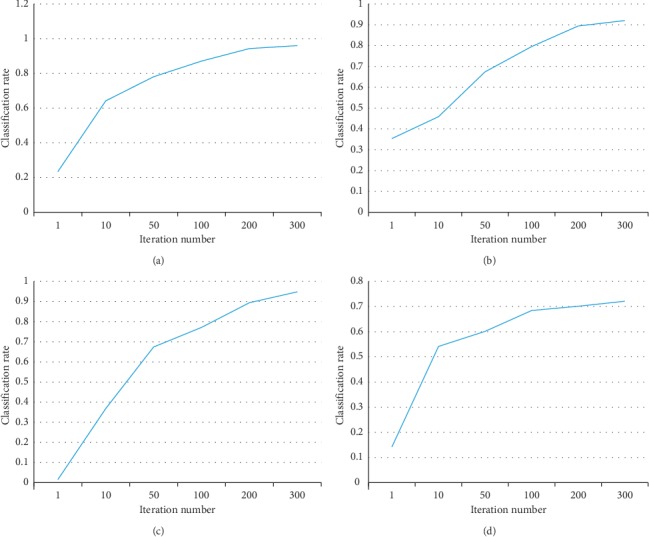
Convergence curves. (a) SkyFinder. (b) Multilingual text-English. (c) FERET face. (d) Property price.

Since our method is an iterative algorithm, it is important to study the convergence of the algorithm. We plot the curve of classification rate with regard to different iteration numbers. The results are shown in Figure 4. From this figure, we have the following observations:

1. In this figure, we can see that for all the benchmark data sets, more iterations always lead to better classification rates. The reason for this phenomenon is that our method updates the inputting neighbors together with the deep learning model parameters. Thus, more iterations give a better estimation of the neighbors, which train a better model.
2. However, we also observe that when the iteration number is larger than 100 (for SkyFinder and property price data sets) or 200 (for multilingual text and FERET face datasets), the performance improvement seems stable. This indicates the convergence of the iterative algorithm.

Remark: we also discuss the possible parameters affecting the convergence as follows:

1. Since our optimization is based on the ADMM algorithm, the ascent/descent step size is an effector that controls the speed of ascent/descent. A lager step size usually results in a faster convergence.
2. Our method's convergence is also affected by the gradient function. This method is also an EM-like algorithm; thus, the convergence is also affected by the slow convergence nature of the EM algorithm, due to the gradient function. Potential solutions include using the conjugate gradient, or the modified Newton's gradient.

#### 3.4.3. Computational Cost

In this section, we discuss the computational cost of the training time of the algorithm. For the four benchmark data sets, we show the training time of our algorithm in Table 1. The following phenomena can be derived from the table:

- The running time for all the four data sets varies according to the size of the training set and the data time. For example, the largest data set, SkyFinder, which contains 90,000 images, our algorithm takes the longest running time of over 8,000 seconds, while for the small data set of property price, the training completes within 400 seconds.
- The higher dimensional data usually consume more training time. The images as the two-dimensional data are more costly than the sequence data as the one-dimensional data. This is natural since the CNN model of the higher dimensional data conducts filtering over the higher dimensional data and the cost is exponentially compared to the lower-dimensional data.
- The overall running time of all the data sets for the proposed algorithm is acceptable. The longest running time is shorter than three hours. This computational cost is reasonable for training a good quality model.

## 4. Conclusion

In this paper, we proposed a novel deep learning framework. Different from existing deep learning models which only take the instances of an input data point, the proposed model can take both the input data point instances and the neighboring data points for the classification of the given input data point. Precisely, we estimate a classification map for each neighboring data point and apply a max-pooling operation to the classification maps of the neighbor to represent the neighbors. Moreover, the classification maps are based on the previous trained deep CNN model. The neighbor classification maps and the CNN model parameters are updated iteratively in an iterative algorithm. Thus, the proposed method is called deep iterative neighborhood. Compared to traditional deep learning methods, the proposed method achieved significant improvement over the classification tasks of benchmark data sets.

## Figures and Tables

**Table 1:** Computational cost of training time.

Data set	Training time (second)
SkyFinder	8674
Multilingual text	1194
FERET face	2687
Property price	311

## Data Availability

All the data sets used in this paper to produce the experimental results are publicly accessed online.
